# Risk assessment of African swine fever transmission by spray-dried porcine plasma in piglet feed and the effect of UV irradiation treatment as an additional safety step

**DOI:** 10.3389/fvets.2025.1463720

**Published:** 2025-09-19

**Authors:** Clazien J. de Vos, Lourens Heres, Aldo Dekker, Manon Swanenburg, W. Meindert Pelser, Jacob Post, Marcel M. Hulst

**Affiliations:** ^1^Wageningen Bioveterinary Research, Wageningen University and Research, Lelystad, Netherlands; ^2^Sonac/Darling Ingredients, Son, Netherlands

**Keywords:** African swine fever, spray-dried porcine plasma, quantitative microbial risk assessment, UV treatment, safety, log_10_ reduction factor

## Abstract

The increase of African swine fever (ASF) outbreaks worldwide has raised concerns about the feeding of spray-dried porcine plasma (SDPP) to pigs. The processing of blood into SDPP should thus guarantee sufficient inactivation of ASF virus (ASFV) to render a safe product. The objective of this study was to evaluate (i) the required level of inactivation if blood of ASF-infected pigs would be processed into SDPP and fed to piglets, and (ii) the additional safety achieved if UV treatment is applied to plasma before spray-drying. A quantitative microbial risk assessment (QMRA) model was built to assess the infection probability (*P_inf_*) of weaned piglets fed with SDPP produced from blood collected from a single ASF-infected herd. The inactivation of ASFV by UV treatment was quantified using a mobile, laboratory-scale “Cold Pasteurization” apparatus (Lyras inc, Aalborg, Denmark). Porcine plasma spiked with blood collected from pigs experimentally infected with ASFV was irradiated with different doses of UV-C and the log_10_ reduction factor (LRF) calculated. An average LRF of 2.2 was achieved by the highest dose of UV-C irradiation applied (~137 Joule/m^2^). QMRA model results indicate that an LRF of 5 needs to be achieved during processing to arrive at a median value of *P_inf_* < 0.01, i.e., less than 1 out of 100 ASF-infected batches resulting in new infections. With an LRF of 8, also the 95th percentile value of *P_inf_* is < 0.01. These results were compared to reported LRF values of spray-drying and dry storage of SDPP, which varied between 5.2 and 11.1. Applying UV-C irradiation as an additional step in SDPP production thus provides extra safety guarantees as the combined inactivation levels of spray-drying, dry storage and UV treatment are likely to result in an overall LRF ≥ 8, implying a very low risk of new ASF infections (median *P_inf_* 7.3 × 10^−6^; 95th percentile 1.6 × 10^−3^). The QMRA model did not account for the probability that ASF-infected pigs are unintendedly processed into SDPP. This probability is low if SDPP is not sourced from pigs in ASF-infected areas, therewith further reducing the ASF infection risk of SDPP.

## 1 Introduction

African swine fever (ASF) is a viral hemorrhagic disease of pigs caused by ASF virus (ASFV), an icosahedral double-stranded DNA virus and the sole member of the *Asfarviridae* family (1, 2). ASFV is endemic in sub-Saharan Africa, where it is maintained in a sylvatic cycle between warthogs and soft ticks of the genus *Ornithodoros* (3). Transmission in European wild boar and domestic pigs is primarily due to direct contact or through ingestion of infected meat products, either by feeding pigs with animal by-products or waste products (swill feeding) or by improperly disposed human waste scavenged by wild boar (1, 3, 4). However, also contaminated fomites and vegetative products, such as cereals and bedding materials, contribute to ASF transmission (5–7). In domestic pigs, ASF is a highly lethal disease resulting in huge economic impact in affected countries. Since the introduction of ASFV genotype II in Georgia in 2007, the geographical distribution of ASF has increased tremendously with the disease now being widely present in Europa and Asia, and also affecting countries in Oceania and the Caribbean (8, 9).

Spray-dried porcine plasma (SDPP) is used worldwide as feed-ingredient for livestock, fish, and pets. SDPP is produced from blood collected from slaughtered pigs by separating plasma from the red blood cell fraction (which includes leucocytes and platelets), after which the plasma is spray dried. A more detailed description of the production process of SDPP is given by Blázquez et al. (44). The resulting protein-rich powder is a highly nutritional and bioactive feed ingredient which is used in starter-diets (11, 12). The use of SDPP in the diet of post-weaning piglets has a positive effect on the health and weight gain of the animals which is attributed to the presence of antibodies. Several studies showed that supplementation with SDPP reduced the severity and length of postweaning diarrhea in piglets, indicating that SDPP may be a favorable feed ingredient and an alternative for antibiotics and Zink oxide (11, 13).

Feeding animal-derived products to animals entails a risk of disease transmission if the raw materials are harvested from infected animals. For the production of SDPP, this risk is reduced by only collecting blood of clinically healthy pigs and by heat treatment during the production process, resulting in (partial) inactivation of pathogens if present. Infected animals can, however, be missed at slaughter if the animals do not show clinical symptoms, or if (mild) clinical symptoms are overlooked or not reported. Then, blood contaminated with pathogens may be included unintendedly in the production process of SDPP. This is illustrated by the presence of genomic material of endemic viruses in SDPP (14). The remaining infectivity after production of SDPP depends on the initial concentration of pathogens in the collected blood and the physical conditions (temperature, pH and duration) of the production process. In SDPP production plants the plasma prepared from raw blood may be alkalized to a pH of 9.8 before being spray-dried at temperatures of minimally 80°C with a residence time varying from 10 to 60 s depending on the industrial spray-dryer installation. Inactivation is also pathogen-dependent, with some pathogens being more resistant to alkaline conditions and high temperatures.

ASFV remains infectious for long periods under ambient conditions in protein-rich environments like meat, blood and other pig-derived products (2, 15, 16). Treatment with relatively high temperatures is needed to completely destroy infectivity of ASFV in these matrices (9). Although spray-drying of alkalized plasma in a laboratory spray-dryer installation inactivated the infectivity of spiked ASFV by more than 99%, still infectious ASFV particles could be rescued from experimentally produced SDPP (17). This was confirmed in a later study (18), where, however, all samples were negative after the subsequent 14-day storage at 20°C which is a common control step in commercial production. Due to denaturation and gelling of the plasma proteins, applying a higher pH and/or temperatures above 80°C in industrial spray-dryers is technically not feasible. Moreover, denaturation of proteins would also nullify the beneficial properties of the SDPP when used as a feed ingredient. As an alternative, UV-C irradiation could be used to further reduce infectivity of ASFV in SDPP. Blázquez et al. (17) demonstrated that UV-C irradiation of SDPP spiked with ASFV prior to spray-drying reduced the infectivity significantly without losing the beneficial properties of SDPP.

The increase of ASF outbreaks worldwide has revived concerns about the feeding of SDPP to pigs. Although the majority of ASFV outbreaks in Europe is in wild boar populations, spill over to domestic pig herds is occurring on a regular basis (19, 20). As a consequence, it cannot be completely ruled out that blood with a high ASFV load may be collected unintendedly from domestic pigs and is used for production of SDPP in case ASFV has been introduced into a free area but has not been detected yet, i.e., during the high-risk period. The different processing steps of the blood should thus guarantee sufficient inactivation of ASFV to render a safe product. The objective of this study was to evaluate (i) the level of inactivation needed in the production of SDPP if ASF-infected pigs would be slaughtered unintendedly and their blood processed into SDPP and fed to piglets, and (ii) the additional safety achieved if UV treatment is applied to plasma before spray-drying. A quantitative microbial risk assessment (QMRA) model was built to assess the infection probability of weaned piglets fed with SDPP produced from blood collected from an ASF-infected herd, and subsequently the level of inactivation needed to achieve an acceptable risk of transmission. The inactivation of ASFV by UV treatment was quantified under laboratory conditions. A mobile, laboratory-scale “Cold Pasteurization” apparatus (Lyras inc, Aalborg, Denmark) able to irradiate plasma with a defined dose of UV-C light was used to study the kinetics of ASFV inactivation in porcine plasma spiked with blood collected from pigs experimentally infected with ASFV. Implementation of such a “Cold Pasteurization” apparatus prior to industrial spray-drying is technically feasible, cost-effective and could reduce the risk of transmission of ASFV via feed and feed ingredients. In this paper, the results of the QMRA model and the UV-C inactivation experiments for ASFV are presented.

## 2 Materials and methods

### 2.1 Quantitative microbial risk assessment

**Figure 1:**
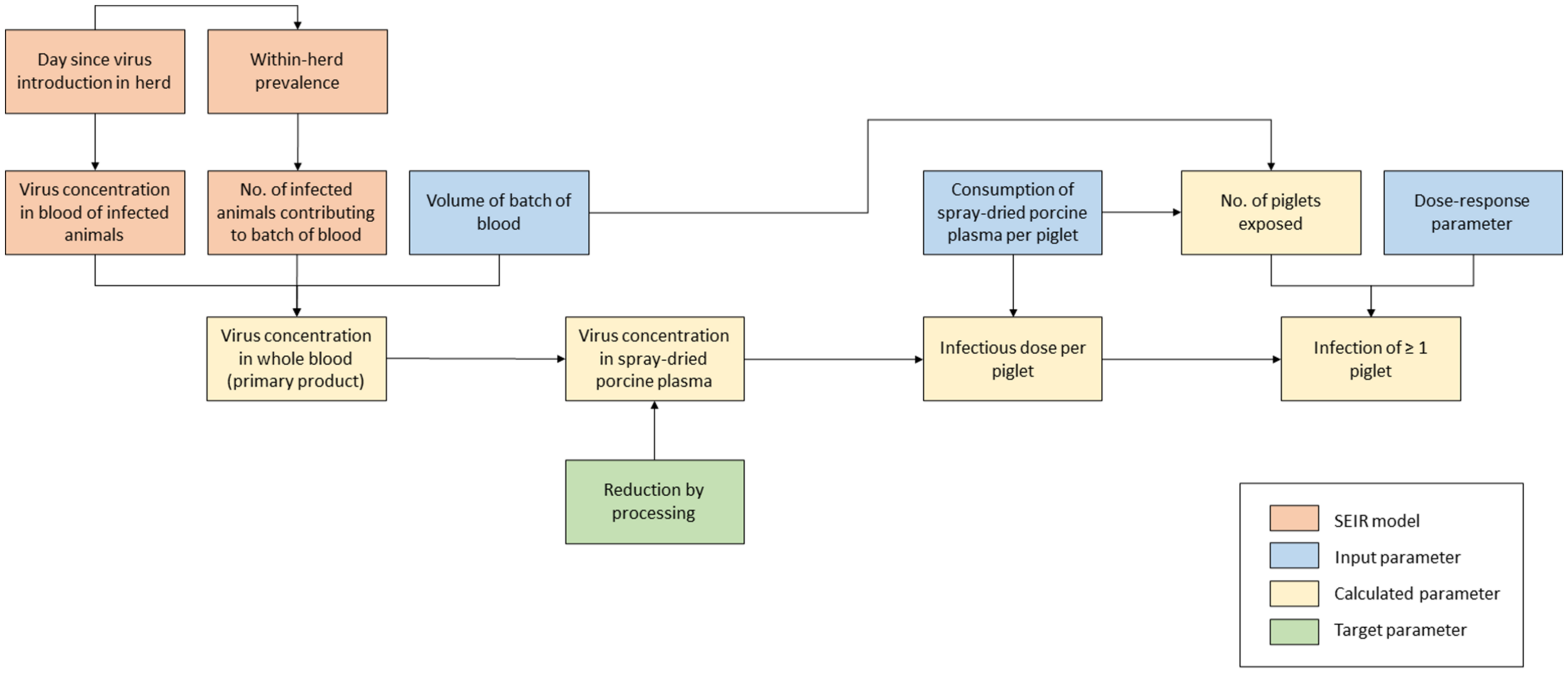
Schematic outline of model calculations to assess the infection risk of plasma powder in weaning feed.

A QMRA model was built to evaluate the infection risk of SDPP if ASF-infected pigs would be slaughtered unintendedly and their blood processed into SDPP. In this model, three main steps can be distinguished (Figure 1):

1. The viral load in blood and SDPP derived from slaughtered animals if ASF infection would be present in a pig herd;
2. The exposure of individual piglets and the number of piglets exposed to ASF-contaminated SDPP;
3. The probability that this exposure will result in new ASF infections.

The model was used to evaluate the log_10_ reduction factor (LRF) of the viral load that should be achieved during the production process of SDPP to guarantee its safety for use in weaning feed for piglets. In this study, we assumed that a probability of new ASF infections due to slaughter of an infected herd ≤1% is an acceptable level of risk.

The QMRA model is a stochastic model built in Excel and @Risk 8.2.1 (21). For each scenario, 10,000 iterations were run to account for uncertainty in model input parameters. Model results are given as median values and 90% uncertainty intervals (bounded by the 5^th^ and 95th percentile values).

#### 2.1.1 Modeling assumptions

The viral load in blood derived from slaughtered fattening pigs is highly dependent on the prevalence of infection in the pig population. We assumed that ASF-infected animals will only be slaughtered and processed in the high-risk period, i.e., before first detection of disease in a newly infected area. Therefore, the viral load was calculated assuming a single infected fattening herd delivering pigs to the slaughterhouse just before the disease would have been detected in this herd. This implies a worst-case scenario as the number of infectious animals in the herd will have reached the maximum level possible without being detected yet.

We assumed that the blood of all ASF-infected animals would be collected by a single processing plant and that it would be processed in a single batch of SDPP. To estimate the ASFV concentration in SDPP, we accounted for dilution of the concentration when mixing the blood of infected animals with blood from non-infected animals. Furthermore, we accounted for the possibility that the ASFV concentration in blood plasma does not equal the ASFV concentration in whole blood due to preference of the virus to connect with either the red blood cell fraction or plasma.

The expected number of new ASF infections due to consumption of contaminated SDPP depends on the exposure of piglets, i.e., the amount of ASFV ingested, and the oral dose–response relationship, i.e., the relation between the ingested dose and the probability of infection, with the probability of infection increasing when higher amounts of virus are ingested (22, 23). In the model calculations, we considered the total viral load ingested during the post-weaning period as a single dose. The dose–response relationship was inferred from infection experiments in which the animals were orally inoculated with the virus.

In the model, viral loads are given in TCID_50_ (tissue culture infectious doses). Input parameters considering viral loads or virus concentrations were sourced from scientific literature and experimental data and were sometimes given in HAD_50_ (hemadsorbing doses) rather than TCID_50_. We assumed both measures to be equivalent. When titers were based on PCR, they were given as TCID_50_eq (equivalents of tissue culture infectious doses).

The amount of blood collected from slaughtered animals was expressed in volumes (liters or milliliters), and virus concentrations in these products were therefore expressed in TCID_50_/mL. Quantities of plasma powder and weaning feed, on the other hand, were expressed as weights and given in grams or kilograms. In the calculations, we assumed liters to equal kilograms and milliliters to equal grams.

#### 2.1.2 Model calculations

##### 2.1.2.1 Viral load in SDPP

A deterministic SEIR model was used to calculate the viral load in whole blood sourced from fattening pigs that were delivered by an infected herd, by calculating (1) the expected number of infected animals in the herd at the day before detection ($Ni_{\text{batch}}$) and (2) the virus concentration (log_10_ TCID_50_) in the blood of each infected animal at the day before detection ($VC_{\text{anima}l_{i,t}}$). To estimate the day of detection, two threshold levels were used: one for the number of animals that died from the infection ($\mathrm{Th}r_{\text{dead}}$) and one for the number of animals showing clinical signs ($\mathrm{Th}r_{\text{clin}}$). Detection was assumed to occur if any of the two thresholds is exceeded. The deterministic SEIR model is described in more detail in Supplementary File 1.

Based on the results of the SEIR model, the ASFV concentration (log_10_ TCID_50_/mL) in a batch of whole blood ($VC_{\text{blood}}$) is calculated as:


(1) $$VC_{\text{blood}}=\log_{10}\frac{\sum_{i}^{Ni_{\text{batch}}}VC_{\text{anima}l_{i,t}}}{Na_{\text{batch}}}$$

Where $VC_{\text{anima}l_{i,t}}$ is the virus concentration (TCID_50_/mL) in the blood of infected animal *i*, dependent on the number of days post infection (dpi) *t*, $Ni_{\text{batch}}$ is the total number of infected animals processed in the batch (here equal to the number of infected animals in a single herd or production unit at the day before detection) and $Na_{\text{batch}}$ is the total number of animals processed in a single batch of blood. $Na_{\text{batch}}$ was estimated by dividing the volume of blood (L) processed in a single batch at the processing plant ($V_{\text{batch}}$) by the volume of blood (L) derived from a single animal ($V_{\text{blood}}$).

The ASFV concentration in blood plasma (log_10_ TCID_50_/mL) before further processing into SDPP ($VC_{\text{plasma}}$) is then calculated as:


(2) $$VC_{\text{plasma}}=VC_{\text{blood}}-RV_{\text{plasma}}$$

Where $RV_{\text{plasma}}$ is the reduction (or increase if its value is negative) of virus concentration in plasma as compared to whole blood after separating the plasma from the red blood cell fraction.

The ASFV concentration in SDPP ($VC_{\text{SDPP}}$) (log_10_ TCID_50_/g) is subsequently calculated as:


(3) $$VC_{\text{SDPP}}=VC_{\text{plasma}}+VI_{\text{SDPP}}-RV_{\text{process}}$$

Where $VI_{\text{SDPP}}$ is the increase of virus concentration in SDPP compared to plasma due to a reduction of volume (assuming that all virus will be retained in the dry fraction), and $RV_{\text{process}}$ is the reduction in virus concentration achieved by the processing conditions of spray-drying. $VI_{\text{SDPP}}$ is calculated as:


(4) $$VI_{\text{SDPP}}=\log_{10}(V_{\text{plasma}}/V_{\text{SDPP}})$$

Where $V_{\text{plasma}}$ is the volume of plasma (L) sourced from 1 L blood and $V_{\text{SDPP}}$ is the weight of SDPP (kg) that can be produced from 1 L whole blood.

##### 2.1.2.2 Exposure of piglets to SDPP

The exposure of individual piglets depends on the ASFV concentration in SDPP ($VC_{\text{SDPP}}$) (log_10_ TCID_50_/g) and the amount of SDPP consumed.

The infectious dose per piglet (*ID*) (log_10_ TCID_50_) is calculated as:


(5) $$\mathrm{ID}=VC_{\text{SDPP}}\times D\times DI_{\text{piglet}}\times C_{\text{SDPP}}$$

Where *D* is the number of days piglets are fed with weaning feed containing SDPP, $DI_{\text{piglet}}$ is the daily intake of weaning feed by a single piglet (g) and $C_{\text{SDPP}}$ is the inclusion rate of SDPP in weaning feed.

The total number of piglets exposed ($N_{\exp}$) to a single ASF-contaminated batch of SDPP is calculated as:


(6) $$N_{\exp}=\frac{V_{\text{SDPP}}\times V_{\text{batch}}}{D\times DI_{\text{piglet}}\times C_{\text{SDPP}}}$$

##### 2.1.2.3 Expected number of new ASF infections

Assuming an exponential dose–response model, the probability for an individual piglet to get infected by ASF (*Pa*_*inf*_) is calculated as:


(7) $$Pa_{\inf}=1-e^{-(10^{\mathrm{ID}}\times \mathrm{DR})}$$

Where *DR* is the dose–response parameter for ASFV when ingested by feed. The dose–response parameter equals the probability of infection for a single TCID_50_ and is estimated from the infectious dose at which 50% of the animals is expected to be infected ($ID_{50}$) (Table 1). The $ID_{50}$ was estimated from experimental data giving the infection probability at different inoculation doses (Supplementary File 2).

**Table 1:** Input parameters used in the quantitative risk model.

Input parameter	Description	Value^a^	Unit	Source
Input parameters SEIR model for within-herd transmission				
*β*	Transmission parameter	RiskPertAlt (2.5%, 0.58, 50%, 1.17, 97.5%, 1.75)	day^−1^	(24)
*T_lat_*	Latent period	5	days	(24)
*T_inf_*	Infectious period	RiskNormal (4.5, 0.75, RiskTruncate(0))^b^	days	(24)
*T_inc_*	Incubation period	RiskPertAlt (2.5%, 2.4, 50%, 4.4, 97.5%, 6.4)	days	(31)
*P_clin_*	Proportion of animals developing clinical signs (morbidity)	1	—	Assumption given high morbidity rate (32)
*P_dead_*	Proportion of animals dying (case fatality rate)	1	—	Assumption given high mortality rate (32)
*Thr_clin_*	Detection threshold based on number of animals with clinical signs	25	animals	Assumption
*Thr_dead_*	Detection threshold based on number of animals that died from infection	15	animals	(27)
*A_fat_*	Average number of fattening pigs per fattening herd	2,645	animals	(33)
*U_fat_*	Number of production units on the fattening farm	10	units	Assumption
Parameters on African swine fever virus in blood				
$VC_{\text{anima}l_{i,t}}$	Virus concentration in the blood of an infected animal *i* on dpi *t* (log_10_ TCID_50_/mL)	RiskLognorm (Mean, SD) (values used are given Supplementary Table S3-1). Based on experimental data from Vlasova et al. (35)		
$RV_{\text{plasma}}$	Reduction of virus concentration after separating plasma from red blood cell fraction	RiskPert (−0.43, 0.95, 2.62)	log_10_	Experimental data WBVR (unpublished)
Parameters for dose–response model				
*ID*_50_	Infectious dose at which 50% of the animals is infected	RiskPertAlt (2.5%, 5.48, 50%, 6.36, 97.5%, 7.23)	log_10_ TCID_50_	Based on experimental data from Niederwerder et al. (37)
*DR*	Dose–response parameter	$\ln(2)/10^{ID_{50}}$	TCID_50_^−1^	Calculated
Production and consumption parameters				
$V_{\text{batch}}$	Average volume of one batch of whole blood (primary product)	130,000	liter	(38)
$V_{\text{blood}}$	Volume of blood derived from a single slaughtered animal	3.5	liter	Sonac/Darling Ingredients
$V_{\text{plasma}}$	Volume of plasma sourced from 1 L blood	0.6	liter	Sonac/Darling Ingredients
$V_{\text{SDPP}}$	Weight of SDPP that can be produced from 1 L blood	0.055	kg	Sonac/Darling Ingredients
*D*	Number of days that piglets are fed weaning feed containing SDPP	14	days	(59)
$DI_{\text{piglet}}$	Daily feed intake of a single piglet	278	g	Sonac/Darling Ingredients
$C_{\text{SDPP}}$	Inclusion rate of SDPP in weaning feed	0.05	—	(59)

a Probability distribution functions as given in the Excel Add-in @Risk. For more information see https://help.palisade.com/v8_2/en/@RISK/Function/Function-Reference.htm.

b Sampled values of the normal distribution were truncated at a minimum value of 0.

The probability that at least one piglet is infected with ASF (*P_inf_*) is then calculated as:


(8) $$P_{\inf}=1-e^{-(N_{\exp}\times 10^{\mathrm{ID}}\times \mathrm{DR})}$$

The expected number of infected piglets (*N_inf_*) from one batch of blood plasma can be calculated assuming a binomial distribution:


(9) $$N_{\inf}=N_{\exp}\times Pa_{\inf}$$

#### 2.1.3 Input parameters

Input parameters needed for the QMRA model were (1) transmission parameters for the SEIR model, (2) parameters on ASFV in blood, (3) the dose–response model, and (4) production and consumption parameters for SDPP. An overview of all input parameters is given in Table 1.

##### 2.1.3.1 SEIR model

To estimate the course of infection in an infected herd with the SEIR model, information was needed on the latent period ($T_{\mathrm{lat}}$), infectious period (*T_inf_*), and the daily rate of transmission (transmission parameter *β*). In the model, values for within-pen transmission were used (24), as these were considered to be most representative for the first phase of infection in a herd (Table 1). The impact of both higher transmission rates (25), lower transmission rates (between-pen transmission as estimated by Guinat et al. (24)), and transmission parameters estimated from field data (26) were explored in what-if scenarios (Table 2).

**Table 2:** Overview of what-if scenarios explored with the quantitative risk model.

No.	Scenario	Parameter changed	Default value^a^	New value^a^	Source
WI-1A	Transmission_A	*β*	RiskPertAlt (2.5%, 0.58, 50%, 1.17, 97.5%, 1.75)	RiskPertAlt (2.5%, 0.16, 50%, 0.61, 97.5%, 1.06)	Between-pen estimates by Guinat et al. (24)
WI-1B	Transmission_B	*β*	RiskPertAlt (2.5%, 0.58, 50%, 1.17, 97.5%, 1.75)	RiskUniform (0.7, 2.2)	(26)
		*T_lat_*	5	RiskUniform (5.8, 9.7)	(26)
		*T_inf_*	RiskNormal (4.5, 0.75, RiskTruncate(0))^b^	RiskUniform (4.5, 8.3)	(26)
WI-1C	Transmission_C	*β*	RiskPertAlt (2.5%, 0.58, 50%, 1.17, 97.5%, 1.75)	RiskPertAlt (2.5%, 0.96, 50%, 2.62, 97.5%, 5.61)	(25)
		*T_lat_*	5	Gamma (19.2, (6.08/19.2))	(25)
		*T_inf_*	RiskNormal (4.5, 0.75, RiskTruncate(0))^b^	Gamma (22.7, (9.15/22.7))	(25)
WI-2	Threshold levels for detection	$\mathrm{Th}r_{\text{clin}}$	25	15	Assumption
		$\mathrm{Th}r_{\text{dead}}$	15	5	(27)
WI-3	Disease symptoms (morbidity and mortality)	$P_{\text{clin}}$	1	RiskBeta (17 + 1, 18–17 + 1)	(34)
		$P_{\text{dead}}$	1	RiskBeta (17 + 1, 18–17 + 1)	(34)
WI-4	Number of production units on fattening farm	$U_{\mathrm{fat}}$	10	3	Assumption
WI-5	Virus concentration in blood of infected animals	$VC_{\text{anima}l_{i,t}}$	RiskLognorm (Mean, SD) (values used are given in Supplementary Table S3-1)	RiskLognorm (Mean, SD) (values used are given in Supplementary Table S3-2)	(36)
WI-6	Virus retained in plasma	$RV_{\text{plasma}}$	RiskPert (0.09, 1.12, 2.62)	0	Worst-case assumption
WI-7	Infectious dose	$ID_{50}$	RiskPertAlt (2.5%, 5.48, 50%, 6.36, 97.5%, 7.23)	4	Based on minimum infection dose given by Niederwerder et al. (37)
WI-8	Volume of batch of blood	$V_{\text{batch}}$	130,000,000	50,000,000	(38)

a Probability distribution functions as given in the Excel Add-in @Risk. For more information see https://help.palisade.com/v8_2/en/@RISK/Function/Function-Reference.htm.

b Sampled values of the normal distribution were truncated at a minimum value of 0.

The number of clinical animals on each day and the cumulative number of dead animals were used to estimate the day that infection would be detected in the herd. Threshold levels for detection were set at 15 animals for dead pigs ($\mathrm{Th}r_{\text{dead}}$) (27) and 25 animals for pigs with clinical signs ($\mathrm{Th}r_{\text{clin}}$) in order to obtain a high-risk period at the farm level that is in accordance with field observations (5, 28–30). The effect of lower threshold levels was explored in a what-if scenario (Table 2).

To estimate the number of clinical and dead animals, information was needed on the incubation period ($T_{\mathrm{inc}}$), and the morbidity and mortality rate ($P_{\text{clin}}$ and $P_{\text{dead}}$). In the model calculations, a rapid onset of disease was assumed with an average $T_{\mathrm{inc}}$ of 4.4 days (31). $P_{\text{clin}}$ and $P_{\text{dead}}$ were both set at 100%, as experimental infections with ASFV strains of genotype II mostly result in morbidity and mortality rates up to 100% in both inoculated and contact animals (32). The effect of a slightly lower morbidity and mortality rate (28) was explored in a what-if scenario (Table 2).

Herd size ($A_{\mathrm{fat}}$) was based on the Dutch 2022 situation, with an average of 2,645 fattening pigs per herd (33). We assumed that production of fattening pigs was based on an all-in all-out system, i.e., all fattening pigs of a single production unit are delivered to the slaughterhouse at the same time. The SEIR model accounted for transmission in a single production unit only. In the default calculations, the number of production units on the farm ($U_{\mathrm{fat}}$) was set to 10. The number of production units in the pig herd was varied in a what-if scenario (Table 2).

##### 2.1.3.2 ASFV in blood

Infected pigs can have very high ASFV concentrations in blood ranging from 6 to 8 log_10_ HAD_50_/mL (31, 34). Only few studies report on virus levels in whole blood of infected animals during the course of infection, i.e., the virus concentration on each dpi ($VC_{\text{anima}l_{i,t}}$). For the QMRA model, default values on virus levels in whole blood were derived from Vlasova et al. (35). In this study, pigs were inoculated with different ASFV strains (all genotype II strains isolated in 2013 in the Russian Federation) using different inoculation doses. We assumed that low doses were most representative for natural infections and calculated the average virus concentration of 6 inoculated pigs for each dpi (Supplementary File 3). Virus titers on days without observations were interpolated based on the virus titers observed on the last measurement before and the first measurement after these days. No measurements were performed by Vlasova et al. (35) after day 19 since all animals had died from the disease by day 20. A gradual decrease of virus titers after day 19 was assumed based on data from Post et al. (36). In this experiment, some animals survived for a longer period and retained virus titers of 4–6 log_10_ TCID_50_eq/mL up till 27 days after infection. The pigs in the experiment by Post et al. (36) were, however, inoculated with Netherlands '86, which is a genotype I strain that is probably less representative for the current genotype II virus circulating in Europe, Asia and the Caribbean. The full dataset from the experiment by Post et al. (36) was used in a what-if scenario (Table 2; Supplementary File 3).

Unpublished data from an animal experiment conducted in the facilities of Wageningen Bioveterinary Research (WBVR) were used to estimate the ASFV reduction when separating plasma from the red blood cell fraction ($RV_{\text{plasma}}$). For 26 samples, the ASFV titer (TCID_50_eq/mL) of infected pigs was determined in both EDTA blood and plasma using PCR. Log_10_ differences in titer between EDTA blood and plasma varied from −0.43 to 2.62, with a mean value of 0.95, indicating that the virus concentration in plasma is on average ~1 log_10_ lower than in blood (Table 1). As a worst-case scenario, we assumed no virus reduction when separating plasma from the red blood cell fraction in the what-if analysis (Table 2).

No input data were collected for the reduction in virus concentration achieved when processing plasma into SDPP ($RV_{\text{process}}$), as the aim of the model was to evaluate the LRF needed for safe application of SDPP in weaning feed. Therefore, the value of $RV_{\text{process}}$ was varied from 0, which mimics the situation without any reduction during processing, to 8, which mimics a reduction in virus concentration of 8 log_10_ TCID_50_ due to processing. Model results of all scenarios were evaluated to assess the LRF that should be achieved during the production process of SDPP to guarantee its safety for use in weaning feed for piglets.

##### 2.1.3.3 Dose–response model

To estimate the dose at which 50% of the animals is expected to become infected ($ID_{50}$), information on the infection probability at different exposure doses is required. Although several experimental studies provided information to estimate the $ID_{50}$ of ASFV (Supplementary File 2), only one study had evaluated the dose–response relationship for exposure to ASFV by ingestion of feed (37). A generalized linear model with a logit link and a binomial error distribution was used to fit a dose–response model to these data and the mean $ID_{50}$ for oral ingestion was estimated at 6.4 log_10_ TCID_50_ (Table 1). Niederwerder et al. (37) estimated a minimum infectious dose of 4 log_10_ TCID_50_ based on their observations. This value was used as an estimate for the $ID_{50}$ in a what-if scenario (Table 2).

##### 2.1.3.4 Production and consumption parameters

The average volume of a single batch of whole blood processed into SDPP ($V_{\text{batch}}$) was assumed to equal 130,000 L (38). The impact of lower production volumes was explored in a what-if scenario (Table 2).

The volume of blood derived from a slaughtered animal ($V_{\text{blood}}$) varies between 3 and 4 L and was set at 3.5 L in the default calculations. One liter of blood yields 600 mL plasma ($V_{\text{plasma}}$). From this, 55 g SDPP can be produced ($V_{\text{SDPP}}$).

We assumed an inclusion of 5% SDPP in weaning feed ($C_{\text{SDPP}}$) and intake of this feed by weaned piglets for a 2-week period (*D* = 14 days). Daily administration of weaning feed ($DI_{\text{piglet}}$) was set at 278 g.

#### 2.1.4 Uncertainty analysis

##### 2.1.4.1 Sensitivity analysis

Uncertain input parameters in the QMRA model were parameterized using input probability distributions. To evaluate the impact of these uncertain input parameters on model results, Spearman rank correlation coefficients between these input parameters and the probability that at least one piglet will be infected (*P_inf_*) were estimated using the sensitivity analysis tool of @Risk. The sensitivity analysis was performed for the simulation run in which the reduction in virus concentration achieved during processing ($RV_{\text{process}}$) resulted in an estimated median *P_inf_* < 0.01.

##### 2.1.4.2 What-if scenarios

To further evaluate the impact of uncertain input parameters on model results, several what-if scenarios were run with the model in which the values for one or more input parameters were changed and the impact on model results evaluated. The what-if scenarios are described in Table 2.

### 2.2 Inactivation of ASFV by UV treatment

#### 2.2.1 Processing of uninfected and ASFV-infected blood to plasma

For UV-C irradiation experiments conducted in this study, raw porcine blood was collected at a regular slaughterhouse in the Netherlands and separated in a production plant from which two batches of 60 L of fresh (unconcentrated) plasma was taken. Briefly, per 1,000 mL of blood 80 mL of a 10% w/v trisodium citrate solution was added on top as anti-coagulation and the blood was cooled to 4°C and centrifuged for 25 min at 1,500×*g* to remove the red blood cell fraction (39). The plasma was alkalized to pH 9.8 by slowly adding small volumes of NaOH solution (1.65 N = 6.6% w/v) under constant agitation. The fresh plasma was kept at 4°C during transport to the WBVR laboratory facility.

To spike the plasma with ASFV, blood of pigs infected with ASFV genotype II strain Armenia/07 was used (40). The blood was collected as slaughter waste from five pigs of an unvaccinated control group of a “vaccination-challenge” animal trial conducted at the animal facilities of WBVR and approved by the Animal Welfare Body of Wageningen University and Research. After culling of the pigs, ASFV-contaminated blood was collected and directly processed to plasma in the same way as the 60-L batches of slaughterhouse blood, except that the contaminated plasma was frozen in 0.25-L portions at −80°C directly after removal of the red blood cell fraction by centrifugation (i.e., the plasma was not alkalized to pH 9.8 before storage). Aliquots of the ASFV-contaminated plasma were frozen separately at −80°C for PCR analysis and titration (see below). Just before the start of each UV-C irradiation run, 1.25 L of ASFV-contaminated plasma was thawed on ice to spike the 60 L batches of slaughterhouse plasma (see below).

#### 2.2.2 UV-C irradiation of plasma batches spiked with ASFV

A mobile, laboratory-scale UV-C “Cold Pasteurization” apparatus, the Raslysation™ Polaris (Lyras inc, Aalborg, Denmark), was used for UV-C irradiation of the plasma. For detailed technical information about the features and operational procedures of this installation we refer to the manufacturer.^1^ A graphic description of the installation is provided in Supplementary File 4. Just before an UV-C irradiation run was started, 60 L of uninfected plasma of pH 9.8 and temperature 4°C was spiked by slowly adding 1.25 L of ice-cold ASFV-contaminated plasma in small portions under constant agitation. The pH of the plasma remained pH 9.8 after spiking. The 61.25 L of spiked plasma was poured into the reservoir of the installation and the volume of plasma needed to fill all physically separated modules of light-permeable spiral tubes was pumped in the irradiation cassette containing the modules. Each module consists of two concatenated spiral tubes placed on top of each other. On both sides of each spiral tube six UV-C lamps are positioned. The intensity of irradiation of the plasma by these two lamps in each spiral tube is adjustable (programmable). During a run the plasma is pumped from the reservoir through the spiral tubes to the exhaust. Samples can be collected after different levels of exposure to irradiation at two different sample ports (port 1 after the first spiral tube and port 2 after the second spiral tube), resulting in a set of plasma samples irradiated with a defined dose in Joule/m^2^. Note that samples collected at port 2 received twice the dose the samples collected at port 1. Furthermore, the plasma in the reservoir which is not pumped through the irradiation cassette is not irradiated. Two identical UV-C irradiation runs were conducted with 61.25 L ASFV-spiked plasma. During each run three different levels of irradiation intensity were set, which in combination with the two sampling ports resulted in six different levels of UV exposure tested (Table 3). Between the two runs, the installation was disinfected by rinsing with 1% w/v NaOH solution followed by multiple rinses with demineralized water. Before starting the UV-C irradiation an aliquot of the 61.25 L ASFV-spiked plasma batch in the reservoir was frozen directly on dry-ice and stored at −80°C. During the runs, collected irradiated plasma samples were labeled with consecutive numbers corresponding with the time (min) of collection. Numbered samples were divided in aliquots and frozen directly on dry-ice before being stored at −80°C. Similarly, at the end of each run, a “non-irradiated” sample of the ASFV-spiked plasma was collected from the reservoir. In Table 3 an overview of all samples collected during the two runs, with their dose of irradiation (Joule/m^2^), is provided. Note that the “time of collection” represents the time in minutes the plasma was present in a module of spiral tubes and does not correspond with the time of irradiation, nor with the dose of UV-C irradiation a sample received.

**Table 3:** Titer of infectious African swine fever virus particles in UV-C irradiated plasma after applying different levels of irradiation with the mobile, laboratory-scale UV-C “Cold Pasteurization” apparatus (Raslysation™ Polaris, Lyras inc, Aalborg, Denmark).

Run number	Type of sample or port-number	Time of collection (min)^a^	Dose (Joule/m^2^)^b^	Titer (log_10_ TCID_50_/mL)^c^	PCR (Ct value)
1	Virus stock	NA^f^	NA	8.0	17.1
1	Start sample^d^	0	0	5.5	22.4
1	1	1	68	3.3	NT^g^
1	2	1	136	2.5	NT
1	1	2	68	3.5	NT
1	2	2	136	2.3	NT
1	1	4	59	3.5	NT
1	2	4	118	2.8	NT
1	1	5	59	3.8	NT
1	2	5	118	2.8	NT
1	1	7	43	4.0	NT
1	2	7	87	3.0	NT
1	1	8	43	3.5	NT
1	2	8	87	3.0	22.9
1	End sample^e^	10	0	4.8	22.4
2	Virus stock	NA	NA	8.0	17.2
2	Start sample	0	0	5.3	22.1
2	1	1	70	3.3	NT
2	2	1	138	2.7	NT
2	1	2	70	3.0	NT
2	2	2	138	2.5	NT
2	1	4	59	3.5	NT
2	2	4	117	3.0	NT
2	1	5	59	3.5	NT
2	2	5	117	2.8	NT
2	1	7	43	3.5	NT
2	2	7	84	3.0	NT
2	1	8	43	3.5	NT
2	2	8	84	3.0	22.5
2	End sample	10	0	5.0	22.6

a Time of collection of irradiated samples from sample port 1 and 2 (note that the “time of collection” represents the time in minutes the plasma was present in a module of spiral tubes and does not correspond with the time of irradiation, nor with the dose of UV-C irradiation a sample received).

b Dose UV-C irradiation registered by the UV-C “Cold Pasteurization” installation.

c Log_10_ TCID_50_/mL; average of two independent titrations.

d Start sample: sample collected directly after spiking of the plasma with ASFV (not irradiated).

e End sample: sample of “non-irradiated” spiked plasma collected at the end of each run (i.e., 10 min after the start of each run).

f Not applicable.

g Not tested.

#### 2.2.3 Titration and qPCR analysis of plasma samples spiked with ASFV

After thawing of aliquots of frozen ASFV-contaminated plasma samples on ice, samples were titrated by end point-dilution using porcine alveolar macrophage cultures (PAMs) as described by Carrascosa et al. (41). Titrations in the lab were performed without knowledge of the dose of irradiation each sample received. To avoid coagulation in the PAM cultures during growth, 20-fold dilutions of plasma samples in culture medium were used as start-dilution and further diluted in 10-fold steps. After 4 days of growth, PAMs infected with ASFV were detected by immunostaining as described by Wensvoort et al. (42) and log_10_ titers were calculated as median tissue culture infectious dose per mL (log_10_ TCID_50_/mL) using the Reed–Muench method (43). Duplicate aliquots of all samples were titrated, and average titers (*n* = 2) were calculated. Duplicate aliquots of samples of the ASFV-contaminated plasma used for spiking (virus stock), the 61.25 L of spiked plasma just before irradiation (start sample), the last plasma sample collected in each run (exposed to 84 or 87 J/m^2^), and the non-irradiated samples of the spiked plasma collected from the reservoir at the end of the runs (end sample) were also analyzed by PCR. Isolation of DNA from plasma samples and analysis of these samples using quantitative real-time polymerase chain reaction (qPCR) was performed as described recently by Eblé et al. (44). Average Ct values (*n* = 2) were calculated.

## 3 Results

### 3.1 Quantitative microbial risk assessment

#### 3.1.1 Viral load in blood and SDPP

The median time until detection of an ASF infection in a fattening pig herd (i.e., the high-risk period) is estimated to be 21 days (90% uncertainty interval (UCI): 18–28 days) in the default scenario. At that time, a total number of 79 pigs (90% UCI: 44–119) is or has been infected. Assuming that the pigs from the infected herd will be delivered to the slaughterhouse at the day before detection, the total amount of ASFV in the blood of slaughtered animals originating from the infected herd is estimated at 10.6 log_10_ TCID_50_ (90% UCI: 9.2–12.6 log_10_ TCID_50_). This results in a median virus concentration of 2.4 log_10_ TCID_50_/mL in a batch of whole blood (90% UCI: 1.1–4.5 log_10_ TCID_50_/mL), 1.5 log_10_ TCID_50_/mL in liquid plasma (90% UCI: −0.21 to 3.7 log_10_ TCID_50_/mL) and 2.5 log_10_ TCID_50_/g in SDPP (90% UCI: 0.83 to 4.7 log_10_ TCID_50_/g) if the spray-drying process would not result in any reduction of infectivity.

#### 3.1.2 Infection risk of ASF

**Figure 2:**
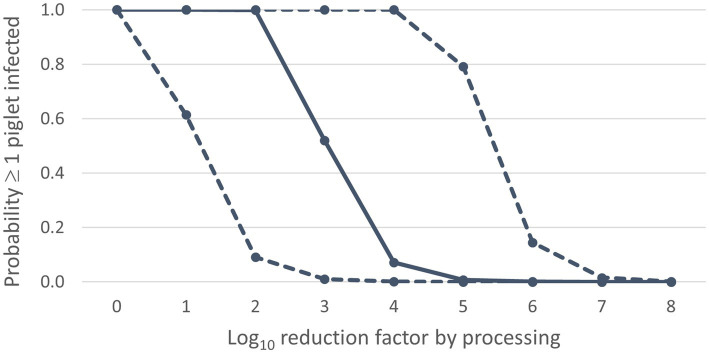
Infection risk of ASF dependent on the virus reduction by processing: median values (solid line) and 90% uncertainty interval (dashed lines) for the probability ≥1 piglet infected (Y-axis) for different values of the log_10_ reduction factor (X-axis).

Results of the default calculations indicate that the probability of ASF infection in weaned piglets due to feeding of contaminated SDPP would be very high when processing of whole blood into SDPP would not result in any virus reduction ($RV_{\text{process}}$ = 0 log_10_) (Figure 2; Supplementary File 5). A virus reduction of ≥ 5 log_10_ is required to achieve a median probability of new infections (*P_inf_*) due to feeding of a contaminated batch of plasma powder ≤0.01, i.e., less than 1 out of 100 contaminated batches is expected to result in new infections. Reducing the virus concentration by 8 log_10_ results in the 95th percentile value of *P_inf_* also being ≤0.01. If the virus concentration is reduced by 5 log_10_, the median value of *P_inf_* is 7.3 × 10^−3^ and the 95th percentile value is 0.79; if the virus concentration is reduced by 8 log_10_, the median value of *P_inf_* is 7.3 × 10^−6^ and the 95th percentile value is 1.6 × 10^−3^ (Supplementary File 5).

In the scenario of no virus reduction ($RV_{\text{process}}$ = 0 log_10_), the feeding of contaminated SDPP is expected to result in 726 infected piglets (median value). The median number of infected piglets is less than 1 in case a 3 log_10_ reduction of the virus concentration is achieved, implying a basic reproduction number (R0) < 1. With a 6 log_10_ reduction, also the 95th percentile value for this output parameter is less than 1 (Supplementary File 5).

#### 3.1.3 Uncertainty analysis

##### 3.1.3.1 Sensitivity analysis

**Figure 3:**
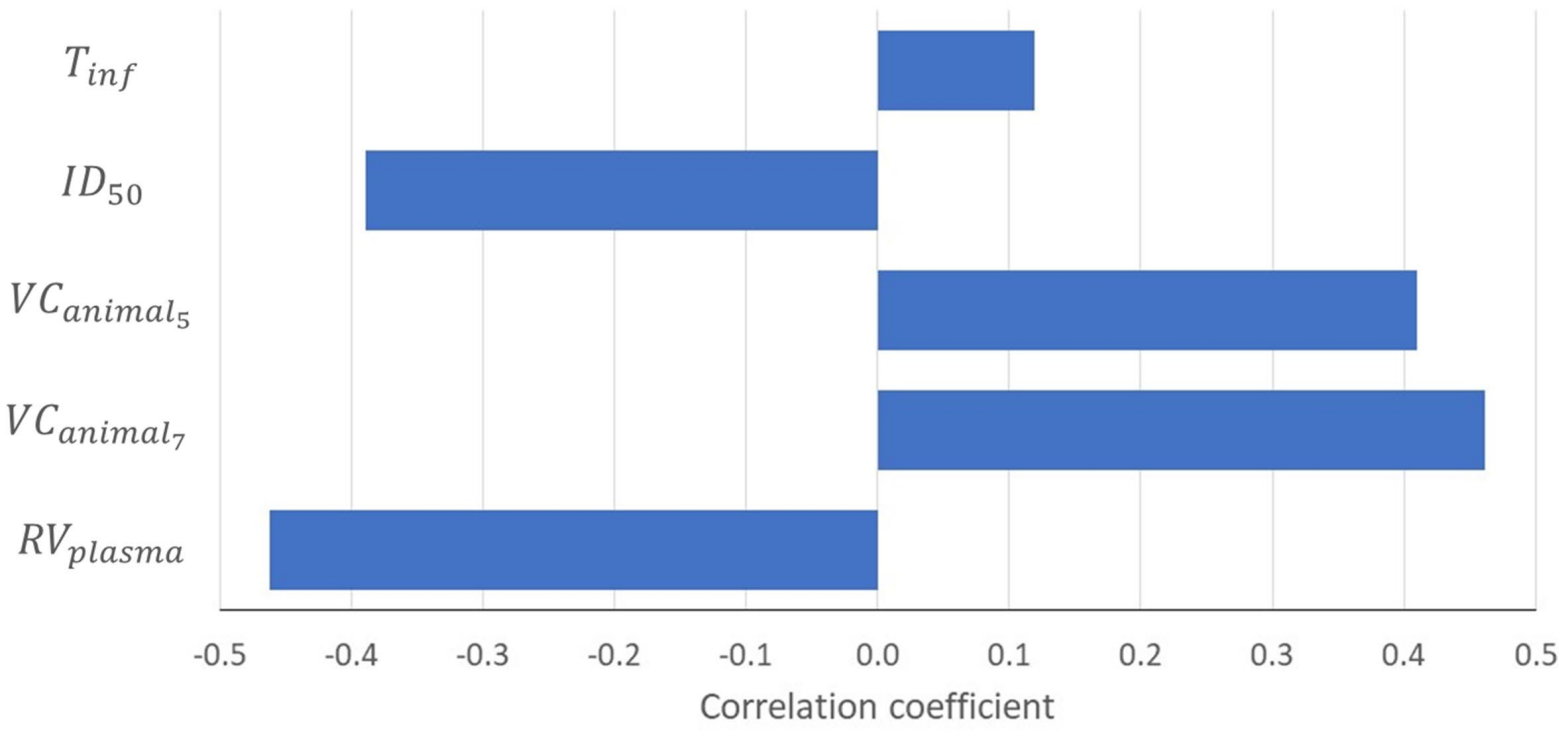
Tornado chart showing the correlation coefficients of input parameters modeled as an uncertainty distribution with the probability that at least one piglet will be infected (*P*_*inf*_). Results are only shown for input parameters with a correlation coefficient ≥|0.1|. $RV_{\text{plasma}}$ = reduction of virus concentration after separating plasma from red blood cell fraction; $VC_{{\text{animal}}_{T}}$ = virus concentration in the blood of an infected animal on dpi t; $ID_{50}$ = infectious dose at which 50% of the animals is infected; *T_inf_* = infectious period.

The sensitivity analysis indicated that the probability ≥1 piglet infected (*P_inf_*) was most sensitive to uncertainty on the reduction of virus concentration after separating plasma from the red blood cell fraction ($RV_{\text{plasma}}$), the virus concentration in blood of infected animals on dpi 5 and 7 ($VC_{\text{animal}i,t=5}$ and $VC_{\text{animal}i,t=7}$) and the estimated infectious dose ($ID_{50}$) (Figure 3). Model results were also sensitive to the infectious period (*T_inf_*), although to a lesser extent. Model results were not sensitive to the transmission parameter (*β*).

##### 3.1.3.2 What-if analysis

**Figure 4:**
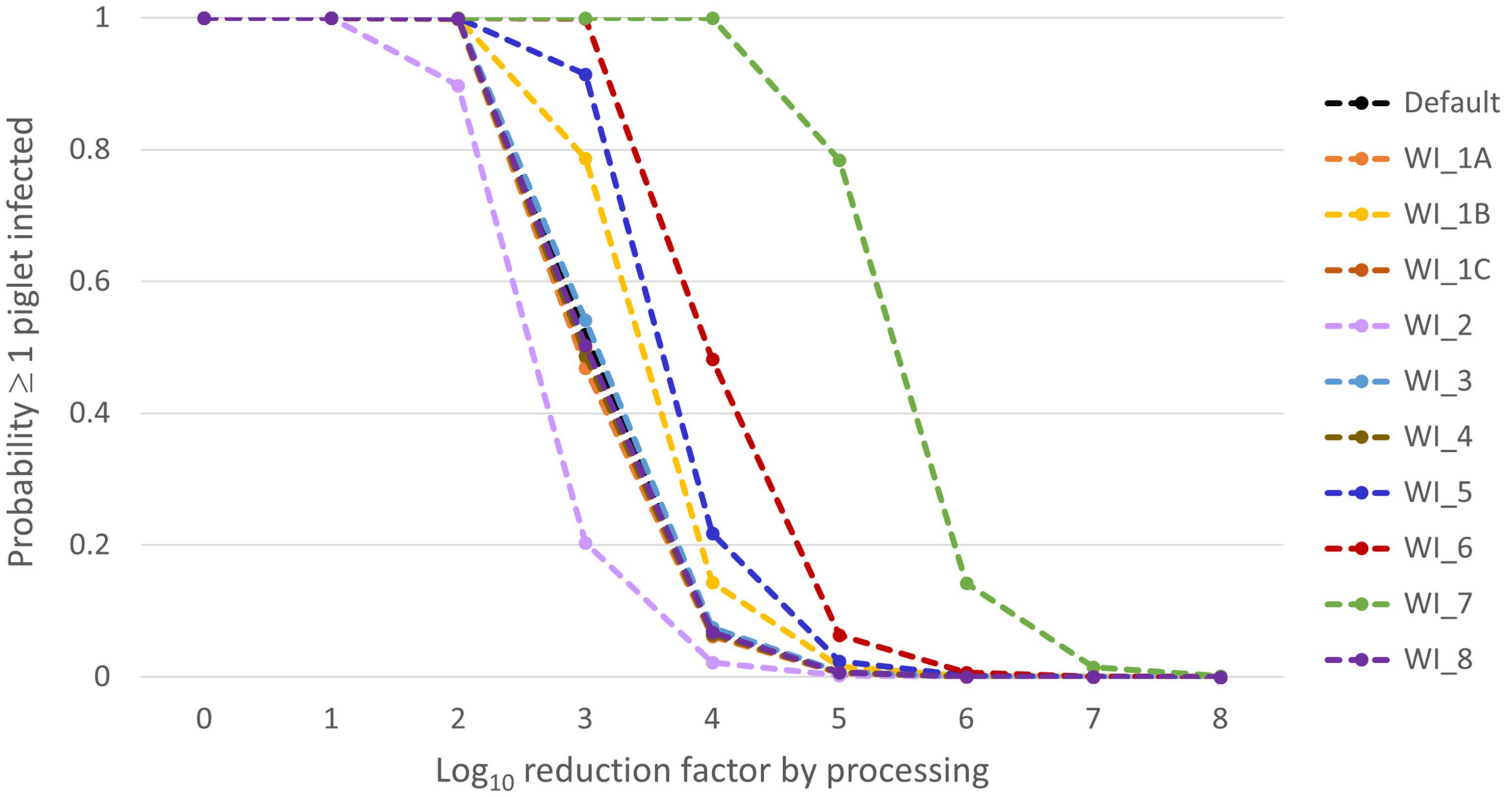
Median results of the what-if scenarios for the probability that at least one piglet will be infected (*P_inf_*) (Y-axis) dependent on the virus reduction by processing (X-axis). The what-if scenarios are described in Table 2.

Results of the what-if analysis are presented in Figure 4. From this figure, it is clear that scenarios WI-6 and WI-7 resulted in a considerable increase of the probability that at least one piglet will be infected (*P_inf_*) compared to the default scenario. In scenario WI-7, the $ID_{50}$ was based on the minimum infectious dose estimated by Niederwerder et al. (37) using the same experiments that we used to estimate the $ID_{50}$ for our default model calculations. This minimum infectious dose was approximately 2.4 log_10_ lower than the $ID_{50}$ value that we had estimated (Supplementary File 2), which resulted in a median 2 log_10_ higher *P_inf_* in this what-if scenario, implying that the LRF achieved during processing of whole blood into SDPP should be increased by 2 to arrive at the same infection risk as in the default scenario. In scenario WI-6, we assumed that separating plasma from the red blood cell fraction would not result in a reduction of the virus concentration. This resulted in a median 1 log_10_ higher *P_inf_* than in the default scenario. Scenarios WI-1B and WI-5 also resulted in a slight increase of *P_inf_* compared to the default scenario. In scenario WI-5 other experimental data were used to model the virus concentration in blood of infected pigs. These results are in accordance with the sensitivity analysis, that also indicated that the model output is highly sensitive to this input parameter. In scenario WI-1B, different values were used for the input parameters of the SEIR model to simulate ASF transmission in the pig herd (*β*, $T_{\mathrm{lat}}$, *T_inf_*). This resulted in a slight increase of *P_inf_*, whereas no effect was observed of changing these input parameters in scenarios WI-1A and WI-1C. This is again in agreement with the sensitivity analysis that indicated that model results were not sensitive to β and only slightly sensitive to $T_{\inf}$. Decreasing the threshold levels for detection (WI-2) slightly reduced *P_inf_* by earlier detection of the infected herd. A slight decrease of morbidity and mortality in infected pigs (WI-3) did, however, not result in an increase of *P_inf_*. Also, the scenarios affecting the size of a production unit on the farm (WI-4) or the volume of a batch of blood processed (WI-8) did not result in a change of *P_inf_*.

### 3.2 Inactivation of ASFV by UV treatment

**Figure 5:**
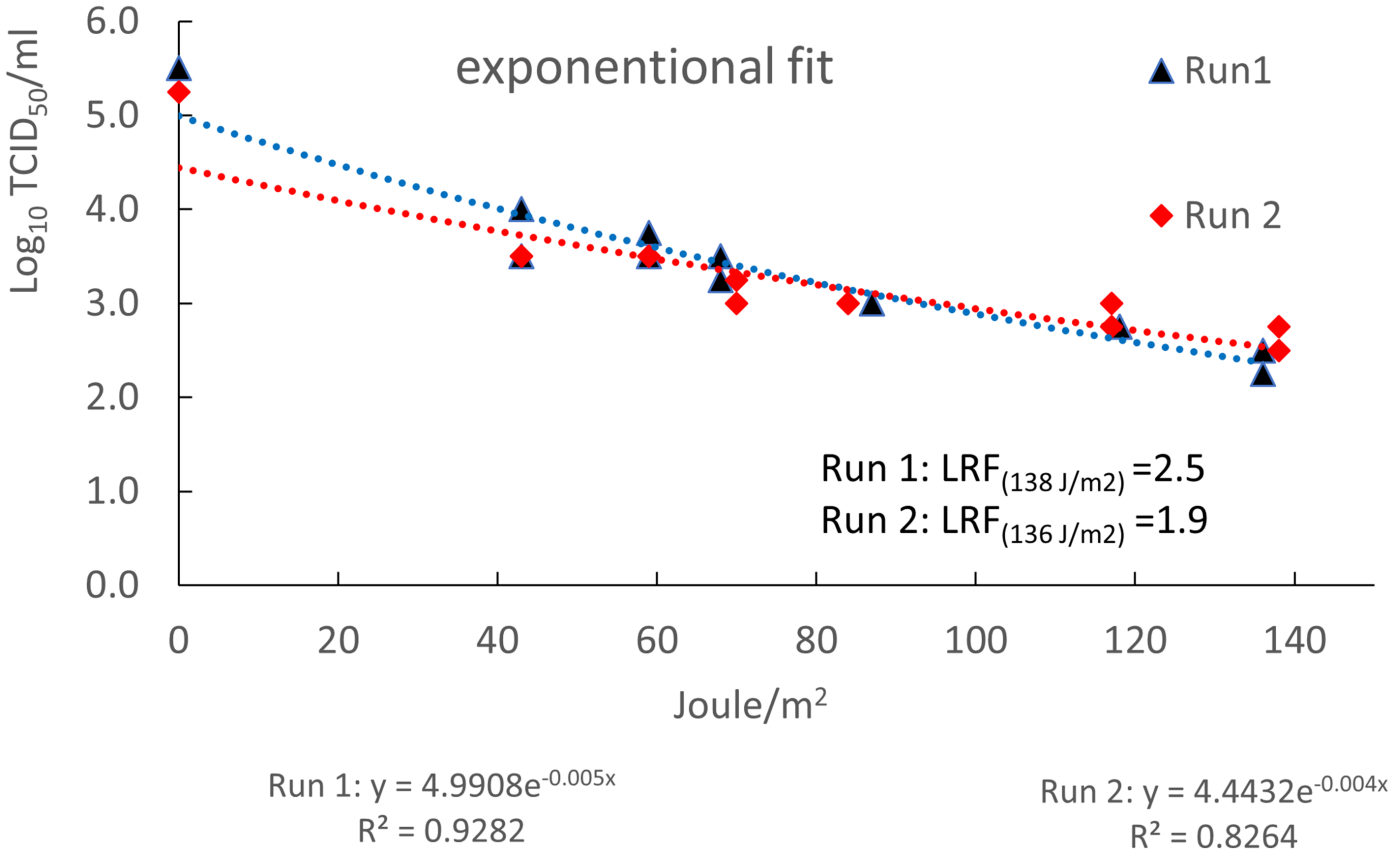
Log_10_ TCID_50_/mL infectious ASFV particles (Y-axis) as function of the dose of UV-C irradiation in Joule/m^2^ (X-axis). Log_10_ reduction factors at the highest dose of UV-C applied were calculated using the formulas of the trendlines displayed beneath the X-axis. *R*^2^: regression coefficients of exponential trendlines.

The titer of the plasma prepared from the blood collected from the ASFV-infected pigs, used for spiking, was 8.0 log_10_ TCID_50_/mL. After spiking the titer of the 61.25 L of plasma measured just before irradiation was 5.5 and 5.3 log_10_ TCID_50_/mL for run 1 and 2, respectively. These titers were 0.8 and 1.0 log_10_ TCID_50_/mL lower, respectively, than the theoretical titer of 6.3 log_10_ TCID_50_/mL calculated from the dilution factor of the virus stock after spiking (Table 3). In each run, samples were collected at sample ports 1 and 2 of the UV-C “Cold Pasteurization” installation after having received different doses of UV-C. In Table 3 the dose of UV-C irradiation (Joule/m^2^) of each sample is displayed with the corresponding log_10_ titer of ASFV as determined by virus titration. No decline in concentration of ASFV-genomes (i.e., a raise in Ct value) was detected by qPCR analysis in the spiked plasma batches subjected to UV-C irradiation by the apparatus (Table 3). This showed that the 1.25 L of ASFV-infected plasma used for spiking was equally distributed in the large batches of 60 L plasma and stayed well dispersed during the period the plasma was present in the light-permeable spiral tubes irradiated with UV-C. Compared to the titer of the plasma measured directly after spiking (start samples), the titers of the non-irradiated spiked plasma collected at the end of run 1 and 2 from the reservoir (end samples) were 0.75 and 0.25 log_10_ TCID_50_/mL lower, respectively. Measured titers of the spiked plasma samples collected at port 1 and 2 were plotted as function of the dose of UV-C irradiation (Figure 5). Exponential trendlines had slightly higher R squared values (*R*^2^) for both run 1 and run 2 than linear trendlines (results not shown for linear trendlines). Based on the equations of the trendlines the calculated LRF achieved after irradiation with the highest dose UV-C applied (~137 Joule/m^2^) was 2.5 for run 1 and 1.9 for run 2. Of the total amount of infectious ASFV particles spiked at the start of the runs, 0.3% (run 1) and 1.3% (run 2) remained infectious after irradiation with ~137 Joule/m^2^ of UV-C light.

## 4 Discussion

A QMRA model was built to estimate the risk of spreading ASF infection by feeding SDPP to piglets, under the assumption that the SDPP would be produced from contaminated blood collected from ASF-infected pigs in a single infected herd. The model can be used to evaluate the level of inactivation that needs to be achieved during the production of SDPP from a disease control perspective, and to compare this with claimed levels of inactivation achieved in the multiple processing steps during production of SDPP to deduce the production process safety guarantees.

The infection risk as calculated by the QMRA model is conditional on the fact that ASF is present in a country or region, but not detected yet, which might result in delivery of ASF-infected pigs to the slaughterhouse. The present work does not consider the probability that pigs from an ASF-infected herd are slaughtered. This probability will in general be low and is dependent on the probability of ASF introduction into the region and the subsequent number of ASF outbreaks during the high-risk period, i.e., before first detection of disease, and therefore also the capacity of surveillance systems in place to detect ASF infection.

Model calculations were based on delivery of ASF-infected pigs by an infected herd just before detection of disease, resulting in high levels of viraemia in the herd and thus also a high viral load in collected blood at the slaughterhouse. This is deemed a worst-case scenario, even more as we did not account for the probability of detection at the slaughterhouse by ante-mortem and post-mortem inspection. Detection of infected animals based on clinical signs (ante-mortem inspection) or pathological lesions (post-mortem inspection) might result in rejection of individual animals or even all animals delivered by the infected herd, lowering the ASF infection risk of SDPP.

EFSA (6) estimated the risk of SDPP contributing to international spread of ASFV very low compared to other matrices such as mash and pelleted compound feed, feed additives and cereals, as SDPP is not sourced from pigs in affected areas and the time window in which animals can be infected without showing clinical symptoms is short. However, EFSA also acknowledged that the risk might be higher in recently affected areas prior to detection of disease when animals in the early stages of infection and without clinical signs might not be detected by ante-mortem or post-mortem inspection at the slaughterhouse. The detection of ASFV RNA in meat seizures at several airports confirms that indeed ASF-infected pigs are slaughtered as fit for human consumption (45). Furthermore, experimental infection showed that 6-month old pigs do not always present with severe clinical symptoms (46).

The infection probability of individual piglets was based on the total infectious dose consumed during a 14-day period. Although the ingestion of virus over a longer period cannot be considered a single dose, the piglets had a repetitive probability of getting infected every day which in the end results in the same probability of infection over the full period considered when an exponential dose–response model is assumed.

We propose that a probability of less than 1% that slaughtered animals of an infected herd would result in new ASF infections is an acceptable level of risk. The QMRA model is, however, flexible to evaluate any other level of risk. We presume that with the veterinary controls in place it is not very likely that pigs from an ASF-infected herd are slaughtered and their blood processed into SDPP, and that it is acceptable that failure of detection would result in a new outbreak once every 100 times this would happen. The QMRA model indicates that on average a 5 log_10_ reduction of the ASF viral load in blood is needed to achieve this acceptable level of risk, and that an 8 log_10_ reduction is required to have 95% certainty that in less than 1% of incidents a new outbreak is initiated.

Uncertainty on the required level of virus reduction needed to achieve an infection probability of less than 1% originated from uncertainty on model input parameters of the QMRA model. The uncertain input parameters affecting model results most were the reduction of ASFV concentration when separating plasma from hemoglobin ($RV_{\text{plasma}}$), the virus concentration in blood of infected animals ($VC_{\text{anima}l_{i,t}}$), and the infectious dose ($ID_{50}$) (Figure 3). A comparison of virus titer (based on PCR results) in both EDTA blood and serum samples of infected pigs was used to estimate $RV_{\text{plasma}}$. Results indicated that ASFV concentrations in serum (and assumingly also plasma) are likely to be lower than in whole blood. This might be explained from the fact that ASFV is a hemadsorbing virus, implying that virus is mostly associated with the red blood cell fraction. A high inter-individual variability was, however, observed, resulting in high uncertainty on this input parameter (Table 1). Assuming equal concentrations of ASFV in plasma and the red blood cell fraction resulted in a median 1 log_10_ higher probability that at least one piglet will be infected (*P_inf_*) compared to the default scenario (Figure 4, WI-6). Limited data were available to estimate the ASFV concentrations in blood of infected pigs during the course of infection ($VC_{\text{anima}l_{i,t}}$). In the default scenario, we used data from experimental studies with ASF genotype II virus strains (35). Uncertainty resulted from observed inter-individual variability in virus titers, as well as limited observation days (Supplementary File 3). We challenged our assumptions in scenario WI-5, where we used data from an experimental study with an ASFV strain of genotype I (36). Results of the what-if analysis indicated a slightly higher ASF infection risk for the genotype I strain (median value of *P_inf_* increased by 0.5 log_10_) (Figure 4, WI-5), which is explained from higher virus titers in blood in the first days after infection (Supplementary File 3). The infectious dose ($ID_{50}$) was estimated from a limited number of animal experiments in which pigs were orally inoculated with virus ‘packed’ in feed (37), resulting in an estimated mean value of 6.4 log_10_ TCID_50_ (Table 1). Using the minimum dose estimated from this study resulted in a median 2 log_10_ higher probability that at least one piglet will be infected (*P_inf_*) (Figure 4, WI-7). A more recent experiment with ASFV-contaminated plasma indicated that the $ID_{50}$ for ASFV when orally inoculated via feed is indeed likely to be high (47). No infection in pigs was reported after daily administration of 4.3 or 5 log_10_ TCID_50_ in feed mixed with contaminated liquid plasma for 14 days. In contrast, Olesen et al. (48) reported 1 out of 4 pigs infected after oral exposure to 5 log_10_ TCID_50_ in blood spiked with ASFV. The differences between these studies could be explained from the matrix in which the virus was presented to the pigs, where virus delivered through liquid might result in infection of target sites of the nasopharynx, including the tonsils, during feeding (37, 47). If we include the exposure of piglets to ASFV in the study of Blázquez et al. (47) in our dose–response model, the median probability of observing no infections is 0.43 (95% UCI: 6.5 × 10^−3^ to 0.87) for a daily administration of 4.3 log_10_ TCID_50_ and 1.4 × 10^−2^ (95% UCI 1.1 × 10^−11^ to 0.48) for a daily administration of 5 log_10_ TCID_50_. The outcome of the higher dose study is thus not very likely based on the ID_50_ estimate that we used in our model, and might be explained from inherent properties of liquid porcine plasma diminishing the infectious capacity of ASFV (47, 49). If indeed porcine plasma would have a protective effect, the infection risk would be even lower than estimated with our model.

Results of the QMRA model are deemed valid for blood sourced from commercial pig production and processed into SDPP in production units of different size. Scenario WI-8 indicated that the production volumes of SDPP are not likely to affect the ASF infection risk. Although the volume of the batch of blood affects the concentration of ASFV in the SDPP, it does not affect the total amount of virus to which piglets are exposed. Also, the size of the production unit on the farm that delivered infected animals to the slaughterhouse (scenario WI-5) is not likely to affect the ASF infection risk for commercial farms, as the number of infected animals is limited by the time until detection rather than the number of animals present in the production unit. This will, however, be different for, e.g., backyard farms, with only a small number of pigs. Similarly, transmission parameters and detection thresholds might differ between commercial farms and backyard farms. Results of the what-if analysis indicated that transmission parameters as such had only limited impact on model results (scenarios WI-1A, WI-1B and WI-1C) as long as detection thresholds are kept the same. Lower detection thresholds (scenario WI-2), however, contributed to a lower ASF infection risk pointing to the importance of early detection, which depends among others on surveillance in place and the level of awareness among farmers and veterinarians. Time until detection is also dependent on the morbidity and mortality among infected animals, which might differ between virus strains. Estimates in the model were all based on the ASF genotype II virus which has been circulating in Eurasia since 2007. Although this virus was highly virulent when introduced, its descendants now show varying virulence (50). Chronic forms of ASF are associated with milder clinical symptoms and lower mortality rates in infected pigs, as well as lower infectious titers in blood (51). A lower viral load in blood will substantially reduce the ASF infection risk of SDPP, although milder clinical symptoms and lower mortality rates might result in an increased number of infected pigs being slaughtered undetected.

Several studies have been performed to evaluate the level of virus inactivation achieved by currently applied or new processing procedures for SDPP for different pig viruses. Most of these studies demonstrated absence of detectable virus in the final product if spray-drying and 14-day dry storage at room temperature are combined, which is standard practice in the production of SDPP (39, 52, 53). The 14-day dry storage period not only contributes to the safety of SDPP by reducing the viral load, but also by allowing the authorities to withhold SDPP batches if a disease suspicion has risen. Similar studies were performed for ASFV. Blázquez et al. (17) estimated a 4.11 ± 0.20 (mean ± SD) log_10_ reduction of ASFV by spray-drying. In a more recent study, a virus reduction between 3.2 and 4.2 log_10_ TCID_50_ was reported for spray-drying at an outlet temperature of 80°C (18). Further inactivation of the viruses was achieved by a 14-day storage period at 20°C, and ASFV could no longer be detected, resulting in an estimated combined inactivation of at least 5.2 log_10_ TCID_50_ (18). This would imply that the combined steps of spray-drying and dry storage at room temperature are sufficient to achieve an acceptable level of risk of ASF infection by SDPP based on median results of the QMRA model. Fischer et al. (54) could no longer detect ASVF after a 2-week storage at room temperature (21 ± 2°C) of SDPP that was spiked with ASFV after spray-drying and concluded that inactivation was at least 5.7 log_10_. The reported inactivation by Fischer et al. (54) is much higher than the inactivation during dry storage reported for other viruses (39). Fischer et al. (54) spiked SDPP with ASFV by adding liquid virus stock to the dry SDPP granules. This might have resulted in a higher inactivation rate as the majority of the spiked ASFV particles were present on the surface of the SDPP granules rather than embedded in the granules, lacking possible natural protection mechanisms against inactivation. However, if indeed these high levels of inactivation could be achieved by dry storage at room temperature, a theoretical 9 log_10_ reduction of ASFV can be achieved by the combined steps of dry spraying and storage, resulting in a probability of at least one new ASF infection (*P_inf_*) < 0.001 even when considering the 95th percentile value (Supplementary File 5). Using a quantitative risk assessment model, Sampedro et al. (55) estimated an 11.1 log_10_ reduction (mean value, 95% confidence interval 10.7–11.5) after spray-drying combined with a 14-day dry storage at 22°C, assuming a decimal reduction time (D-value) of 2 days at this temperature. This reduction would even suffice to reach the Performance Objective of < 1 virus particle in the final product as set by Sampredo et al. (55) given an initial viral load of blood of 10.6 log_10_ TCID_50_ as estimated by our model (Section 3.1.1). However, these estimates are all based on experimental settings, and we do not know if these are equally valid for bulk processing of SDPP. Also, it is not known if the inactivation rates measured for the separate processes can be simply added or that there is a decreasing inactivation rate at lower doses. It is, however, not easy to measure the combined effect of the different steps in an experimental setting, as the amount of virus with which blood can be spiked is limited to a range of 5–8 log_10_ TCID_50_ [this study; (17, 54)]. If the assumption of addition does not hold true, a higher inactivation rate would be required for each of the individual steps. These uncertainties, as well as uncertainties in the QMRA model results, become less relevant if additional inactivation steps are added to the production process of SDPP. UV-C irradiation of plasma before spray-drying could be considered as an additional control step to inactivate ASFV to increase certainty that sufficient levels of ASFV inactivation are reached without affecting the quality of SDPP.

The experiments conducted in this study showed that UV-C irradiation can reduce the concentration of infectious ASFV in plasma by more than 99% under experimental settings. Irradiation of fresh plasma of pH 9.8 with a dose of UV-C light of ~137 Joule/m^2^ in a laboratory-scale “Cold Pasteurization” apparatus achieved an average 2.2 log_10_ reduction of ASFV in two independent experiments. Figure 5 illustrates that the level of inactivation of ASFV was not linearly correlated with the dose applied, but showed an exponential decline, i.e., the inactivation was faster in the beginning and decreased at increasing dosage. This indicates that applying higher doses of UV-C irradiation might not lead to complete inactivation of infectious ASFV in plasma. Part of the infectious ASFV in blood of infected pigs may be trapped in aggregates of cellular debris and/or protein complexes and shielded for UV-C irradiation. In UV-C inactivation experiments in water with several pathogenic bacteria (56) and bacteriophage MS2 (57) it was shown that shielding due to the presence or emerge of aggregates also reduced the efficiency of inactivation by UV-C irradiation.

In a study performed by Blázquez et al. (17) a significant higher log_10_ reduction of ASFV was achieved after UV-C irradiation of ASFV-spiked plasma with a LRF of 4.6 at a dose of 3,000 Joule/L. Results of both studies cannot be easily compared for several reasons. First, Blázquez et al. (17) used a different laboratory-scale apparatus to irradiate the plasma (a SurePure Turbulator™ apparatus, manufactured by SurePure Operation AG, Zug, Switzerland), and the dose of irradiation was expressed in Joule/L plasma instead of Joule/m^2^. Second, in the present study alkalized plasma (pH 9.8) derived from healthy pigs was spiked with pH-neutral plasma prepared from the blood of pigs infected with a virulent ASFV strain, whereas Blázquez et al. (17) spiked pH-neutral plasma derived from healthy pigs with a cell culture-adapted strain of ASFV produced *in vitro*. As reported for other enveloped (e.g., porcine epidemic diarrhea (PED) virus) and naked viruses (e.g., Adenovirus) (39, 58), in our study spiking in alkalized plasma of pH 9.8 resulted in partial inactivation of ASFV infectivity with an estimated LRF of ~0.9 (Table 3). Furthermore, differences in processing between *in vivo* and *in vitro* cells of moieties on the surface of ASFV particles (e.g., glycosyl groups) may also result in a difference in resistance of ASFV particles to UV-C irradiation. Third, in the present study only one passage of the plasma through the apparatus, taking 1 min, is used to achieve the exposure to ~137 Joule/m^2^ and a LRF of 2.2 (see Table 3), whereas in the SurePure Turbulator™ apparatus 7 min of circulation of the plasma through the apparatus was needed to reach the same level of inactivation at an irradiation dose of ~1,375 Joule/L. Despite the differences between the two studies, they both showed that UV-C irradiation reduced the load of infectious ASFV in fresh plasma by at least 99%. Therefore, implementation of UV-C irradiation of fresh plasma prior to spray-drying in SDPP production plants may contribute to a reduced risk of ASFV transmission when feeding SDPP to piglets, and also reduces the risk of other viral and bacterial pathogens harmful for pigs (10).

## 5 Conclusion

The present study used a quantitative risk assessment approach to determine how much virus inactivation is needed to guarantee safety of SDPP when considering the ASF transmission risk. The results show that a high level of inactivation is needed (between 5 and 8 log_10_), which can only be reached by a combination of inactivation steps. Estimated values for the inactivation of ASFV by the combination of spray-drying and dry storage are between 5.2 and 11.1 log_10_. UV-C irradiation of plasma could be used as an additional virus inactivation step for ASFV. This study showed that an average 2.2 log_10_ reduction of ASFV can be achieved with UV-C irradiation (dose of ~137 Joule/m^2^) under experimental settings. UV-C irradiation can be applied flexible in SDPP-production plants, e.g., in case blood is sourced in regions where ASFV is present in the wild boar population and where additional assurances are needed that processing blood into SDPP does not contribute to further spread of the epidemic. The addition of UV-C irradiation in the production process provides these extra guarantees as the combined inactivation levels of spray-drying, dry storage and UV treatment are likely to result in ≥8 log_10_ reduction of the virus load. UV-C irradiation thus provides an extra margin of safety, which makes up for the uncertainty in the QMRA model results on the one hand, and the uncertainty in estimated inactivation achieved by the individual and combined processing steps of SDPP on the other hand. This additional safety might be considered sufficient to authorize the use of SDPP from free pig herds in areas where ASFV is circulating in wild boar populations.

The QMRA framework of this study can also be applied to other porcine viruses in plasma, including both notifiable and endemic viruses. The model will indicate how much virus reduction is needed to obtain an acceptable level of risk, and which input parameters are of critical importance. The reported inactivation from the SDPP production process can then be compared with the required inactivation to assess the safety of SDPP. New viruses will require new input data to parameterize the model. Also, the acceptable level of risk might be different for viruses that are endemic (e.g., PED virus or porcine reproductive and respiratory syndrome (PRRS) virus) compared to viruses of emerging and notifiable porcine diseases like the case of ASFV presented in this study.

## Data Availability

The original contributions presented in the study are included in the article/Supplementary material, further inquiries can be directed to the corresponding author.

## Footnotes

^1^ https://lyras.com/technology-uv-pasteurization/
