# Supplementary material for: Risk assessment of African swine fever transmission by spray-dried porcine plasma in piglet feed and the effect of UV irradiation treatment as an additional safety step
Source: Front Vet Sci. 2025 Sep 19;12:1463720. doi: 10.3389/fvets.2025.1463720 (PMC12492952; doi:10.3389/fvets.2025.1463720)
Supplement: Supplementary file 1 [file Data_Sheet_1.docx]

Supplementary Material

# Deterministic SEIR model

The deterministic SEIR model calculates the expected number of susceptible (S), exposed (E), infectious (I) and removed (R) animals on a daily basis, assuming a fixed number of animals (N) on the farm (i.e. natural births and deaths were ignored). The transmission chain starts with all animals but one being susceptible to the disease, as one animal is assumed to have been exposed to the disease at the first day of the transmission chain. Exposed animals are those animals that have been infected, but are not infectious yet, i.e. they are in the latent phase of infection. Infectious animals are those that shed the virus and can transmit the virus to susceptible herd mates. Removed animals are those that are no longer infectious, either because they have recovered or died. Main input parameters for the SEIR model are the herd size *N*, the transmission parameter β, the length of the latent period ($T_{lat}$, with σ=1/$T_{lat}$) and the length of the infectious period ($T_{inf}$, with γ=1/$T_{inf}$). To calculate the expected number of animals with clinical signs in the herd (*C*), we also included the incubation period ($T_{inc}$, with 𝜌=1/$T_{inc}$), the clinical period ($T_{clin}$, with τ=1/$T_{clin}$), and the morbidity rate (ω=$P_{clin}$) as input parameters. The number of animals incubating the disease (*Y*) was calculated as an intermediate step to calculate the number of clinical animals (*C*). The case fatality rate (μ=$P_{dead}$) was included to calculate the expected number of animals dying from the infection (*D*). Animals were assumed to die at the end of the infectious period.

Values of input parameters are given in Table 1 of the paper. Please note that the herd size *N* was based on the average number of fattening pigs in a production unit, i.e. $N={A_{fat}}/{U_{fat}}$ (value of *N* rounded to the nearest integer). No input was defined for the clinical period ($T_{clin}$). The clinical period was assumed to end at the same moment as the infectious period (death of the animal) and was inferred from the length of the latent, infectious and incubation period as $T_{clin}=T_{lat}+T_{inf}-T_{inc}$. As this could result in negative values for the clinical period in case of a short latent and/or infectious period and a long incubation period, we included a correlation matrix in the model to ensure that if sampled values for the incubation period were high, sampled values for the infectious period would also be high. No correlation with the latent period was included, since the latent period was modelled as a fixed value

The number of animals in each class is calculated on a daily basis (Fig. S1-1). The infection is assumed to start with one exposed animal at day 1, i.e. the initial numbers in each class are: $S=N-1$, $E=1$, $I=0$, $R=0$, $Y=0$, $C=0$ and $D=0$. In calculating the number of animals in each class, we assumed frequency-dependent transmission and homogeneous mixing (Keeling and Rohani, 2008). Estimates for the transmission parameter β were based on within-pen transmission, assuming that within-pen transmission is most representative for the first phase of infection in the herd.

Figure S1-1. SEIR model for disease transmission in a herd.

The change in animal numbers over time for each class is calculated as (Fig. S1-1):

- Susceptible animals: $\frac{dS}{dt}=-\beta\frac{SI}{N}$
- Exposed animals: $\frac{dE}{dt}=\beta\frac{SI}{N}-\sigma E$
- Infectious animals: $\frac{dI}{dt}=\sigma E-\gamma I$
- Removed (=no longer infectious) animals: $\frac{dR}{dt}=\gamma I$
- Incubating animals: $\frac{dY}{dt}=\omega\beta\frac{SI}{N}-\rho Y$
- Clinical animals: $\frac{dC}{dt}=\rho Y-\tau C$
- Dead animals: $\frac{dD}{dt}=\mu\gamma I$

To estimate the day of detection, two threshold levels are used: one for the number of animals showing clinical signs (${Thr}_{clin}$) and one for the number of animals that died from the infection (${Thr}_{dead}$). For each day, the estimated numbers of clinical and dead animals are compared to these threshold levels and detection is assumed to occur att the first day that one of these threshold levels is exceeded. The expected virus concentration in the blood of each infected animal in the herd (i.e. exposed and infectious animals) at the day before detection is subsequently calculated, considering the days post infection (dpi) for each individual animal.

The SEIR model provided the expected number of infected animals at the day before detection (${Ni}_{batch}$) and the ASFV concentration in blood of each infected animal *i*, based on its dpi *t* (${VC}_{{animal}_{i,t}}$) as input to the QMRA model calculations (Eq. 1 in the paper).

## References

Keeling MJ, Rohani P. Modeling Infectious Diseases in Humans and Animals. Princeton University Press, New Jersey, USA (2008).

# Dose-response model

The data from the studies in Table S2-1 were used to fit dose-response models for four different inoculation routes: intramuscular, intranasal, oral in liquid and oral in feed using a generalized linear model (Fig. S2-1). The estimated values of the ${ID}_{50}$ for each route are given in Table S2-2.

Table S2-1. Studies providing information to fit the dose-response relationship for different ASF virus strains and inoculation routes.

| Strain | Inoculation route | Reference |
| --- | --- | --- |
| Georgia 2007 | Oral (in feed) | Niederwerder et al., 2019 |
| Georgia 2007 | Oral (in liquid) | Niederwerder et al., 2019 |
| Malawi | Oral (in liquid) ^a^ | Howey et al., 2013 |
| Malawi | Intranasal^b^ | Howey et al., 2013 |
| Brazil ’78 | Intranasal | De Carvalho Ferreira et al., 2012 |
| Malta ’78 | Intranasal | De Carvalho Ferreira et al., 2012 |
| Netherlands ’86 | Intranasal | De Carvalho Ferreira et al., 2012 |
| Netherlands ’86 | Intranasal | Post et al., 2017 |
| Armenia 2008 | Intranasal^c^ | Pietschmann et al., 2015 |
| Russia Kashino 04/13 | Intranasal | Vlasova et al., 2015 |
| Russia Boguchary 06/13 | Intranasal | Vlasova et al., 2015 |
| Russia K 08/13 | Intramuscular | Vlasova et al., 2015 |
| Lithuania LT14 | Intramuscular | Gallardo et al., 2017 |
| Malawi | Intramuscular | Howey et al., 2013 |

^a^ Described as intraoropharyngeal inoculation in the paper

^b^ Described as intranasopharyngeal inoculation in the paper

^c^ Described as oronasal inoculation in the paper

Table S2-2. Estimated values of the ID_50_ for ASF virus, i.e. the dose at which 50% of the animals is expected to become infected given in log_10_ TCID_50_ for three different inoculation routes^a^.

| Inoculation route | Mean | Lower confidence limit (2.5 percentile value) | Upper confidence limit (97.5 percentile value) |
| --- | --- | --- | --- |
| Oral (in feed) | 6.356 | 5.479 | 7.233 |
| Oral (in liquid) | 1.207 | 0.447 | 1.967 |
| Intranasal | 2.089 | 1.493 | 2.684 |

^a^ It was not possible to estimate the ${ID}_{50}$ for the intramuscular inoculation route, as all pigs at all doses got infected.

Figure S2-1. Relation between inoculation dose (virus titer in log_10_ TCID_50_) and fraction of animals infected. Solid red line: intramuscular application. Dashed blue line: oral (in liquid) application. Dashed green line: intranasal application. Dashed red line: oral (in feed) application. The size of the marker indicates the number of pigs inoculated at that dose.

## References

de Carvalho Ferreira HC, Weesendorp E, Elbers AR, Bouma A, Quak S, Stegeman JA, et al. African swine fever virus excretion patterns in persistently infected animals: a quantitative approach. Vet Microbiol (2012) 160(3-4):327-40. doi: 10.1016/j.vetmic.2012.06.025

Gallardo C, Soler A, Nieto R, Cano C, Pelayo V, Sánchez MA, et al. Experimental Infection of Domestic Pigs with African Swine Fever Virus Lithuania 2014 Genotype II Field Isolate. Transbound Emerg Dis (2017) 64(1):300-304. doi: 10.1111/tbed

Howey EB, O'Donnell V, de Carvalho Ferreira HC, Borca MV, Arzt J. Pathogenesis of highly virulent African swine fever virus in domestic pigs exposed via intraoropharyngeal, intranasopharyngeal, and intramuscular inoculation, and by direct contact with infected pigs. Virus Res (2013) 178(2):328-39. doi: 10.1016/j.virusres.2013.09.024

Niederwerder MC, Stoian AMM, Rowland RRR, Dritz SS, Petrovan V, Constance LA, et al. Infectious Dose of African Swine Fever Virus When Consumed Naturally in Liquid or Feed. Emerg Infect Dis (2019) 25(5):891-897. doi: 10.3201/eid2505.181495

Pietschmann J, Guinat C, Beer M, Pronin V, Tauscher K, Petrov A, et al. Course and transmission characteristics of oral low-dose infection of domestic pigs and European wild boar with a Caucasian African swine fever virus isolate. Arch Virol (2015) 160(7):1657-67. doi: 10.1007/s00705-015-2430-2

Post J, Weesendorp E, Montoya M, Loeffen WL. Influence of Age and Dose of African Swine Fever Virus Infections on Clinical Outcome and Blood Parameters in Pigs. Viral Immunol (2017) 30(1):58-69. doi: 10.1089/vim.2016.0121

Vlasova NN, Varentsova AA, Shevchenko IV, Zhukov IY, Remyga SG, Gavrilova VL, et al. Comparative Analysis of Clinical and Biological Characteristics of African Swine Fever Virus Isolates from 2013 Year Russian Federation. British Microbiology Research Journal (2015) 5:203-215. doi: 10.9734/BMRJ/2015/12941

# Viral load in blood

Table S3-1. Virus concentration in blood of ASF infected animals (log_10_ HAD_50_/cm^3^) (based on data from Vlasova et al., 2015).

| Dpi | 1 | 3 | 5 | 7 | 9 | 11 | 13 | 15 | 17 | 19 |
| --- | --- | --- | --- | --- | --- | --- | --- | --- | --- | --- |
| N | 6 | 6 | 6 | 5 | 4 | 4 | 2 | 1 | 1 | 1 |
| Mean | 1.00 | 2.92 | 5.00 | 6.00 | 5.63 | 6.00 | 5.75 | 6.50 | 7.50 | 7.00 |
| SD | 0.77 | 1.46 | 1.48 | 0.94 | 1.38 | 0.71 | 0.35 | NA | NA | NA |

**Table S3-2.** Virus concentrations in blood of ASF infected animals (log_10_ TCID_50_eq/ml) (based on data from Post et al., 2017)^a^.

| Dpi | 3 | 5 | 7 | 10 | 14 | 17 | 20 | 24 | 27 |
| --- | --- | --- | --- | --- | --- | --- | --- | --- | --- |
| N | 20 | 20 | 20 | 19 | 11 | 6 | 6 | 6 | 6 |
| Mean | 3.31 | 6.39 | 6.35 | 6.16 | 5.84 | 5.71 | 5.53 | 5.46 | 5.33 |
| SD | 2.08 | 0.27 | 0.16 | 0.10 | 0.19 | 0.20 | 0.30 | 0.36 | 0.45 |

^a^ Titers from this study were based on PCR and estimated as TCID_50_eq (equivalents of tissue culture infectious doses). We only used observations if a positive virus isolation was reported at the same point in time.

## References

Post J, Weesendorp E, Montoya M, Loeffen WL. Influence of Age and Dose of African Swine Fever Virus Infections on Clinical Outcome and Blood Parameters in Pigs. Viral Immunol (2017) 30(1):58-69. doi: 10.1089/vim.2016.0121

Vlasova NN, Varentsova AA, Shevchenko IV, Zhukov IY, Remyga SG, Gavrilova VL, et al. Comparative Analysis of Clinical and Biological Characteristics of African Swine Fever Virus Isolates from 2013 Year Russian Federation. British Microbiology Research Journal (2015) 5:203-215. doi: 10.9734/BMRJ/2015/12941

# UV-C “Cold Pasteurization” apparatus: Raslysation^TM^ Polaris (Lyras inc, Aalborg, Denmark)

More detailed information available online at: <https://lyras.com/wp-content/uploads/2022/04/Raslysation-Polaris-1.0-Spec-Sheet-LBH-10_11_22-LS.pdf>.


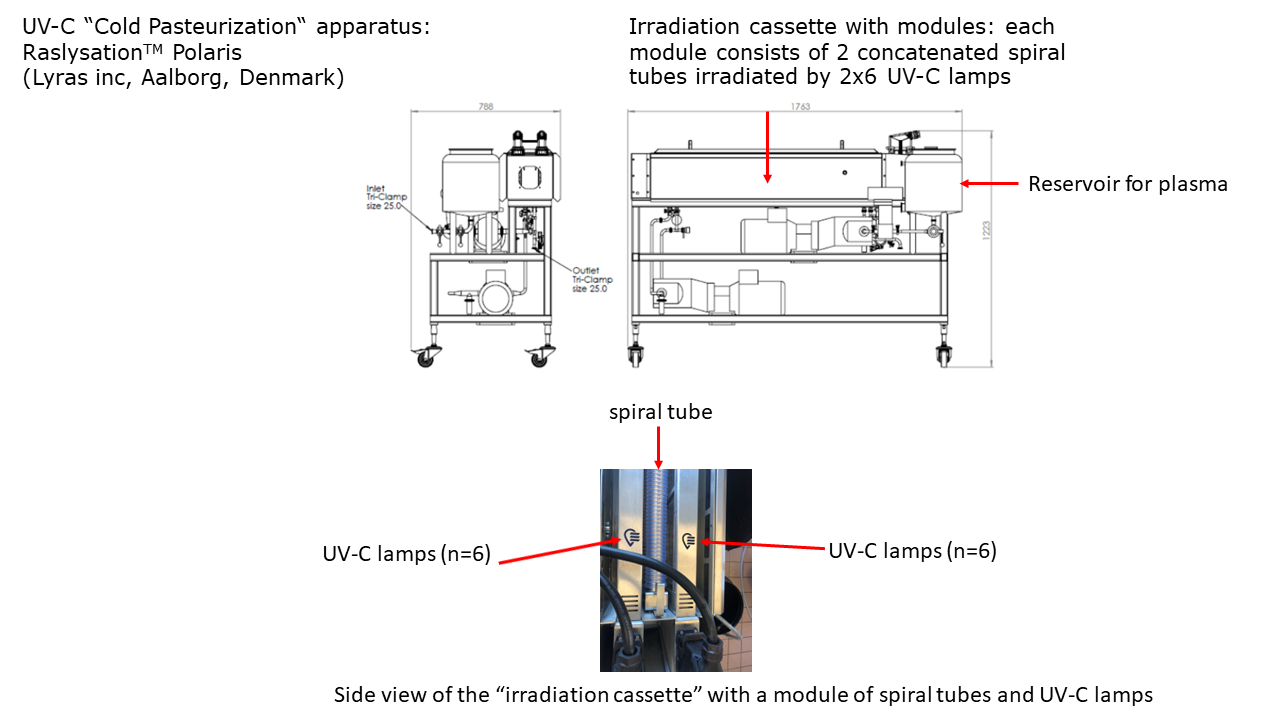


# Results QMRA model

Table 5-1: Infection risk of ASF dependent on the virus reduction achieved by processing plasma into SDPP: median values and 90% uncertainty intervals for the probability ≥ 1 piglet infected ($P_{inf}$), and the expected number of piglets infected ($N_{inf}$). Shading of risk levels: median value of $P_{inf}$ > 0.01 (red); 95^th^ percentile value of $P_{inf}$ < 0.01 (green); otherwise (orange).

| Log_10_ reduction | $\boldsymbol{P}_{\boldsymbol{inf}}$ | | $\boldsymbol{N}_{\boldsymbol{inf}}$ | |
| --- | --- | --- | --- | --- |
|  | Median | 90% UCI | Median | 90% UCI |
| 0 | 1 | 1 – 1 | 726 | 9.52 – 36226 |
| 1 | 1 | 0.61 – 1 | 73 | 0.95 – 12760 |
| 2 | 1 | 0.091 – 1 | 7.3 | 0.10 – 1535 |
| 3 | 0.52 | 9.5E-03 – 1 | 0.73 | 0.010 – 156 |
| 4 | 0.071 | 9.5E-04 – 1 | 0.073 | 9.5E-04 – 16 |
| 5 | 7.3E-03 | 9.5E-05 – 0.79 | 7.3E-03 | 9.5E-05 – 1.6 |
| 6 | 7.3E-04 | 9.5E-06 – 0.15 | 7.3E-04 | 9.5E-06 – 0.16 |
| 7 | 7.3E-05 | 9.5E-07 – 0.016 | 7.3E-05 | 9.5E-07 – 0.016 |
| 8 | 7.3E-06 | 9.5E-08 – 1.6E-03 | 7.3E-06 | 9.5E-08 – 1.6E-03 |
| 9 | 7.3E-07 | 9.5E-09 – 1.6E-04 | 7.3E-07 | 9.5E-09 – 1.6E-04 |
| 10 | 7.3E-08 | 9.5E-10 – 1.6E-05 | 7.3E-08 | 9.5E-10 – 1.6E-05 |
| 11 | 7.3E-09 | 9.5E-11 – 1.6E-06 | 7.3E-09 | 9.4E-11 – 1.6E-06 |
| 12 | 7.3E-10 | 9.5E-12 – 1.6E-07 | 7.3E-10 | 8.2E-12 – 1.6E-07 |
